# AN IMPLEMENTATION BOOSTER TO EMPOWER THE PATIENT AND CAREGIVER: 4MS ADVOCACY TIP SHEET

**DOI:** 10.1093/geroni/igae098.0481

**Published:** 2024-12-31

**Authors:** Betty Napoleon, Mary Zonsius, Elizabeth Zimmerman, Megan Foradori, Mary Dolansky

**Affiliations:** Case Western Reserve University, Cleveland, Ohio, United States; Rush University, Chicago, Illinois, United States; Case Western Reserve University, Cleveland, Ohio, United States; Case Western Reserve University, Cleveland, Ohio, United States; Case Western Reserve University, Cleveland, Ohio, United States

## Abstract

Consumer driven reminders are another way to increase the uptake of evidence-based practices. With that in mind, our patient advocacy council engaged in the development of an Age-Friendly Advocacy Tip Sheet. Patients shared that they were unaware as to how to ensure that they received AFHS 4Ms care. Patients’ views enhance insights into booster strategies for implementation of the 4Ms. It is not only essential for providers to have access to resources to deliver the 4Ms of care, but it is equally important for consumers to have tools to boost 4Ms care. Through multiple iterations, feedback sessions, and on-going qualitative analysis, we supported our Patient Advisory Council to create an Age-Friendly Health Systems Advocacy Tip Sheet to empower older adults to prioritize the 4Ms of care during appointments. This Tip Sheet includes self-advocacy suggestions, information on the 4Ms of care, and a work-sheet style space to list questions and concerns before the visit. There is also a space for note taking during the visit, along with next steps for acting on what matters. A scannable QR Code on the back of the Tip Sheet provides access to additional resources and information on Age-Friendly Health Systems. Feedback from both providers, patients, and other older adults has been overall positive, with remarks such as it is helpful, user-friendly, colorful, easy to understand, and applicable to pre-appointment preparation.

